# Real‐life efficacy and safety of vemurafenib plus rituximab in relapsed or refractory hairy‐cell leukemia: A multicenter Italian retrospective study (HCL‐PG03R)

**DOI:** 10.1002/hem3.70255

**Published:** 2025-11-14

**Authors:** Luca De Carolis, Monia Capponi, Andrea Bernardelli, Andrea Visentin, Gianna Maria D'Elia, Simone Ferrero, Michele Cimminiello, Azzurra Romeo, Barbara Pocali, Luciana Morino, Alessandro Sanna, Ombretta Annibali, Francesca Ricci, Flavio Falcinelli, Alessandro Mancini, Francesco Angotzi, Anna Maria Frustaci, Nilla Maschio, Caterina Stelitano, Alessandro Gozzetti, Riccardo Moia, Maura Nicolosi, Silvia Trappolini, Valerio Leotta, Alessandro Pulsoni, Robin Foà, Carlo Visco, Roberta Murru, Jacopo Olivieri, Livio Trentin, Brunangelo Falini, Enrico Tiacci

**Affiliations:** ^1^ Department of Medicine and Surgery, Center for Hemato‐Oncological Research, Institute of Hematology University and Hospital of Perugia Perugia Italy; ^2^ Hematology Unit, Department of Medicine Azienda Ospedaliera Universitaria Integrata di Verona Verona Italy; ^3^ Department of Medicine, Hematology and Clinical Immunology Branch Padua University School of Medicine Padova Italy; ^4^ Hematology, Department of Translational and Precision Medicine Sapienza University Rome Italy; ^5^ Division of Hematology, AOU “Città della Salute e della Scienza di Torino” Torino Italy; ^6^ Oncology Department UOC Hematology and Stem Cell Transplantation, AOR San Carlo Potenza Italy; ^7^ Division of Hematology and Stem Cell Transplantation Ospedale S.M. Goretti Latina Italy; ^8^ AO di Rilievo Nazionale Antonio Cardarelli UOC Ematologia con Trapianto di Midollo Napoli Italy; ^9^ Hematology Sant'Eugenio Hospital Rome Italy; ^10^ SOD Hematology AOU Careggi Firenze Italy; ^11^ Divison of Hematology University Campus Bio‐Medico Rome Italy; ^12^ Humanitas Cancer Center Division of Hematology Milan Italy; ^13^ Department of Hematology ASST Grande Ospedale Metropolitano Niguarda Milan Italy; ^14^ Onco Hematology, Department of Oncology Veneto Institute of Oncology IOV, IRCCS Padua Italy; ^15^ Hematology Unit Ospedale Bianchi Melacrino Morelli Reggio Calabria Italy; ^16^ Hematology Unit, Azienda Ospedaliera Universitaria Senese University of Siena Siena Italy; ^17^ Division of Hematology, Department of Translational Medicine Università del Piemonte Orientale and Azienda Ospedaliero‐Universitaria Maggiore della Carità Novara Italy; ^18^ SC Ematologia, AOU Città della Salute e della Scienza Torino Italy; ^19^ Hematology Clinic, AOU Marche Ancona Italy; ^20^ UOC Hematology ARNAS Garibaldi‐Nesima Hospital Catania Italy; ^21^ Hematology, Department of Translational and Precision Medicine, S.M. Goretti Hospital Sapienza University Latina Italy; ^22^ Hematology and Stem Cell Transplantation Unit Ospedale Oncologico A. Businco ARNAS “G. Brotzu” Cagliari Italy; ^23^ Division of Hematology and Stem Cell Transplantation, ASUFC Udine Italy

Hairy‐cell leukemia (HCL) is a rare, chronic mature B‐cell neoplasm, usually presenting with cytopenias and splenomegaly at a median age of 55–60 years with a marked male predominance (male‐to‐female ratio ~ 4:1).^1^, ^2^ Standard front‐line chemotherapy with purine analogs (PNAs; cladribine or pentostatin) produces durable complete remissions (CRs) in ~80%–85% of cases and a median relapse‐free survival > 10 years, yet up to 58% of patients eventually relapse and become progressively less sensitive to these myelotoxic and immune‐suppressive drugs.^1^, ^3^, ^4^, ^5^

Discovery of the activating BRAF‐V600E kinase mutation as the founding genetic lesion in >95% of HCL cases,^6^ and of other genetic lesions of BRAF or MAP2K1 in the rare cases negative for BRAF‐V600E,^7^, ^8^ provides both a useful diagnostic marker and a therapeutic target tractable with oral BRAF or MAP2K1 inhibitors.^7^, ^9^, ^10^, ^11^, ^12^, ^13^, ^14^, ^15^, ^16^, ^17^, ^18^, ^19^ In particular, clinical trials of vemurafenib or dabrafenib treatment for a short and fixed duration in HCL relapsed after, or refractory to, PNAs (R/R‐HCL) produced ~90% overall response and ~35% CR rates.^10^, ^11^, ^17^, ^18^ However, all CRs were positive for minimal residual disease (MRD), and relapse of cytopenias usually occurred relatively early (median ≤ 1.5 years) after treatment cessation.

A subsequent academic single‐center Phase 2 trial (HCL‐PG03) on 30R/R‐HCL patients with a median of 3 prior therapies tested vemurafenib (960 mg b.i.d. for a total of 8 weeks, with a 2‐week interval of drug holiday after the first 4 weeks) in combination with rituximab/MabThera (a monoclonal antibody against CD20, which is highly expressed by HCL) given intravenously for eight doses (375 mg/mq every 2 weeks; four doses concomitant to vemurafenib and four sequential). Such a very short chemotherapy‐free regimen led to dramatically improved results: 87% CR rate, 60% MRD‐negativity rate, and a progression‐free survival (PFS) of 78% at a median follow‐up of 37 months, with relatively limited and manageable toxicities.^18^

Here, to validate the HCL‐PG03 single‐center trial results in the real world of routine clinical care, we performed a multicenter retrospective study (HCL‐PG03R) on 54 patients with BRAF‐V600E + HCL (R/R, *n* = 52; newly diagnosed, *n* = 2) who were homogenously treated, at 22 Italian centers between June 2019 and April 2025, with 8 continuous weeks of vemurafenib (960 mg b.i.d.) plus four concomitant and four sequential doses of rituximab (mostly biosimilar: *n* = 50/54 patients, 93%; 375 mg/m^2^ intravenously every 2 weeks), with follow‐up until June 2025.

Patients (Table 1 and Table S1) had a median of two prior treatments (range 0–11), including cladribine in 47/54 (87%) of cases, pentostatin in 17/54 (31%), interferon in 15/28 (28%), rituximab in 16/54 (30%), and BRAF inhibitor‐based therapy in 5/54 (9%). In total, 20% of patients (11/54) were refractory to their last PNA course, including 15% (8/54) primary refractory; 3/54 patients (6%) were refractory to rituximab, and 1/54 (2%) to vemurafenib. The median baseline hemoglobin, neutrophils, and platelets were 11 g/dL, 0.86 × 10⁹/L, and 65 × 10⁹/L, respectively; 8/54 patients (15%) required transfusions of erythrocytes (*n* = 8 patients) and platelets (*n* = 1 patient). Among the 51/54 (94%) non‐splenectomized patients, 26/51 (51%) had palpable splenomegaly, and across the 49/51 evaluated radiologically, the longest splenic diameter measured a median of 14.5 cm (range 7–23). The median bone marrow infiltration by HCL cells was 80% (range: 20%–100%).

**Table 1:** Patients characteristics.

Characteristics	Patients (*n* = 54)
Male sex (%)	46 (85)
Median age (range)	56 years (35–80)
Previous therapies (%)	Median 2 (range 0–11)
Cladribine	47 (87)
Rituximab	17 (31)
Pentostatin	16 (30)
Interferon alpha	15 (28)
Splenectomy	3 (6)
Vemurafenib	1 (2)
Vemurafenib + cobimetinib	1 (2)
Vemurafenib + rituximab	1 (2)
Vemurafenib + cobimetinib + obinutuzumab	1 (2)
Dabrafenib	1 (2)
Zanubrutinib	1 (2)
Moxetumomab pasudotox	1 (2)
Fludarabine	1 (2)
Bendamustine	1 (2)
Refractory to purine analog (%)	11 (20)
Primary refractory to purine analog (%)	8 (15)
Refractory to rituximab (%)	3 (6)
Blood counts pre‐therapy	Median (range)
Hemoglobin, g/dL	11 (6.4−14.7)
Platelets/mmc	65,000 (21,000−710,000)
Neutrophils/mmc	855 (160−2150)
Transfusion requirement (%)	8 (15)
Red blood cells	8 (15)
Platelets	1 (2)
Median HCL infiltration in pre‐therapy bone marrow biopsy (range)	80% (20%–100%)
Splenomegalic pts. by physical examination	26/51 (51%)
Median spleen diameter by radiology^a^	14.5 cm (range 7–23)
Type of rituximab	
Truxima (%)	46 (85.2)
Mabthera (%)	4^b^ (7.4)
Rixathon (%)	3 (5.6)
Ruxience (%)	1 (1.9)
Median time to resolution of cytopenias^c^ in weeks (range)	
Anemia (*n* = 24 pts)	6 (1–11)
Thrombocytopenia (*n* = 38 pts)	2 (1–6)
Neutropenia (*n* = 51 pts)	4 (1–10)
Response to vemurafenib + rituximab (%)	
Complete response	43 (80)
MRD‐negative^d^ MRD‐positive MRD non available	29/43 (67) 11/43 (26) 3/43 (7)
Partial response	4 (7)
Not evaluable	7 (13)
Progression‐free survival (*n* = 54 pts.)	90%
Median follow‐up from the end of treatment	24 months (range 2–62)
Dose density of vemurafenib (i.e., no. of days needed to complete the planned 8 weeks = 56 days of vemurafenib)^e^	Median 56 days (IQR 56–66; range 56–136)
Relative dose intensity of vemurafenib (960 mg b.i.d. for 8 weeks)^e^	Median 95% (IQR 75%–100%; range 14%–180%)
≤50%	5 pts. (10%)
>50% ≤80%	12 pts. (23%)
>80%	34 pts. (67%)

Abbreviations: HCL, hairy‐cell leukemia; IQR, interquartile range; MRD, minimal residual disease; pts., patients.

^a^ Longest spleen diameter in the 51 non‐splenectomized patients radiologically evaluated for splenomegaly (by ecography, *n* = 43; by computed tomography, *n* = 7; or by magnetic resonance imaging, *n* = 1).

^b^ One pt. on truxima had to switch to mabthera due to a moderate‐intensity infusion‐related reaction after the second dose.

^c^ Hemoglobin ≥ 11 g/dL, platelets ≥ 100,000/mmc, and neutrophils ≥ 1500/mmc.

^d^ <0.05% BRAF‐V600E alleles by digital PCR (*n* = 26) or <0.1% HCL cells by flow cytometry (*n* = 3) in the bone marrow aspirate.

^e^ In 51/54 evaluable pts.

Notably, 28% of the 54 patients had active (*n* = 4) or latent (*n* = 11) infections at treatment start, including: ongoing systemic atypical mycobacteriosis (*n* = 1); ongoing large cerebral abscess (*n* = 1); severe bacterial pneumonia improving on antibiotic therapy (*n* = 1); ongoing miliary tuberculosis (*n* = 1; the latter two patients, 65‐year and 80‐year old, were the only two with untreated HCL); HIV infection controlled by antiviral therapy (*n* = 1); and latent tuberculosis (*n* = 1) or hepatitis B virus (*n* = 9) infections requiring antimicrobial prophylaxis.

Hematological recovery was rapid: platelet (≥100 × 10⁹/L), neutrophil (≥1.5 × 10⁹/L), and hemoglobin (≥11 g/dl) thresholds for response^20^ were reached at a median of 2, 4, and 6 weeks, respectively (very similar to the 2, 4, and 4 weeks reported in the trial^18^). In total, 4/54 of patients (7%) had hematological recovery but did not undergo a posttreatment bone marrow biopsy for full response assessment per consensus guidelines (whereby CR requires the absence of HCL cell infiltration recognizable on hematoxylin–eosin staining, without the help of immunohistochemistry)^20^; among the remaining 50 patients, 43/50 (86%) reached a CR (including 2 patients with delayed neutrophil recovery from rituximab toxicity, and 1 with persistent splenomegaly likely unrelated to HCL); 4/50 (8%) reached a partial response (PR), including 1 case whose immediate prior therapy was a course of vemurafenib + rituximab received 86 months earlier in our trial^18^; and the remaining 3/50 patients deceased before completing treatment due to preexisting atypical mycobacteriosis, complications of neurosurgery for preexisting cerebral abscess, and sudden death unrelated to the study drugs or to HCL. Notably, the other 2/4 patients with preexisting active infection (bacterial pneumonia and miliary tuberculosis) reached CR and resolved the infection, which supports vemurafenib + rituximab as a safe and effective treatment to clear leukemia and restore immunity in this clinically challenging scenario, as previously reported.^1^, ^14^ Importantly, a CR was achieved in all evaluable cases refractory to PNA (*n* = 9/9) or rituximab (*n* = 3/3) or previously splenectomized (*n* = 3/3), as well as in most patients (*n* = 4/5) with prior exposure to BRAF inhibitor‐based therapies (delivered 55–56–60–85 months earlier), confirming regimen activity in these relevant subgroups of interest as seen in the trial.^18^ Also similar was MRD‐negativity rate as evaluated in the bone marrow aspirate by BRAF‐V600E droplet digital PCR (Bio‐Rad assay dHsaCP2000028, setting a threshold for MRD positivity of ≥0.05% mutant alleles as used in the trial^18^) and/or standard flow cytometry (sensitivity: ≥0.1% HCL cells): 73% of the CR cases evaluated for MRD were negative (*n* = 29/40), leading to an overall MRD clearance rate of 62% (*n* = 29/47, i.e., including the 4 PRs and the 3 deaths), with only 2 discordant cases (both MRD+ by PCR and MRD− by flow cytometry) among the 28/40 CR cases analyzed with both techniques.

The median follow‐up after starting treatment was 22 months among all 54 patients (range: 1–66 months) and 24 months in the 51 alive patients (range: 5–66 months). At 24 months, PFS was 90% (Figure 1A) and overall survival was 94% (Figure 1B). PFS events were represented by three deaths occurring at ≤3 months (including one death unrelated to HCL or its treatment) and by four relapses (defined by reappearance of cytopenias per consensus guidelines^20^) occurring 13–41 months after CR (*n* = 2) or PR (*n* = 2) (Figure 1C), with PFS being significantly longer after CR (97%) than after PR (75%; log‐rank P‐value 0.005). Among the 43 CR patients, at a median follow‐up of 27 months from treatment start (range 6–66), PFS was longer in MRD‐negative (100%) versus positive (88%) cases (P‐value 0.024; Figure 1D), as also observed in the previous trial.^18^

**Figure 1:**
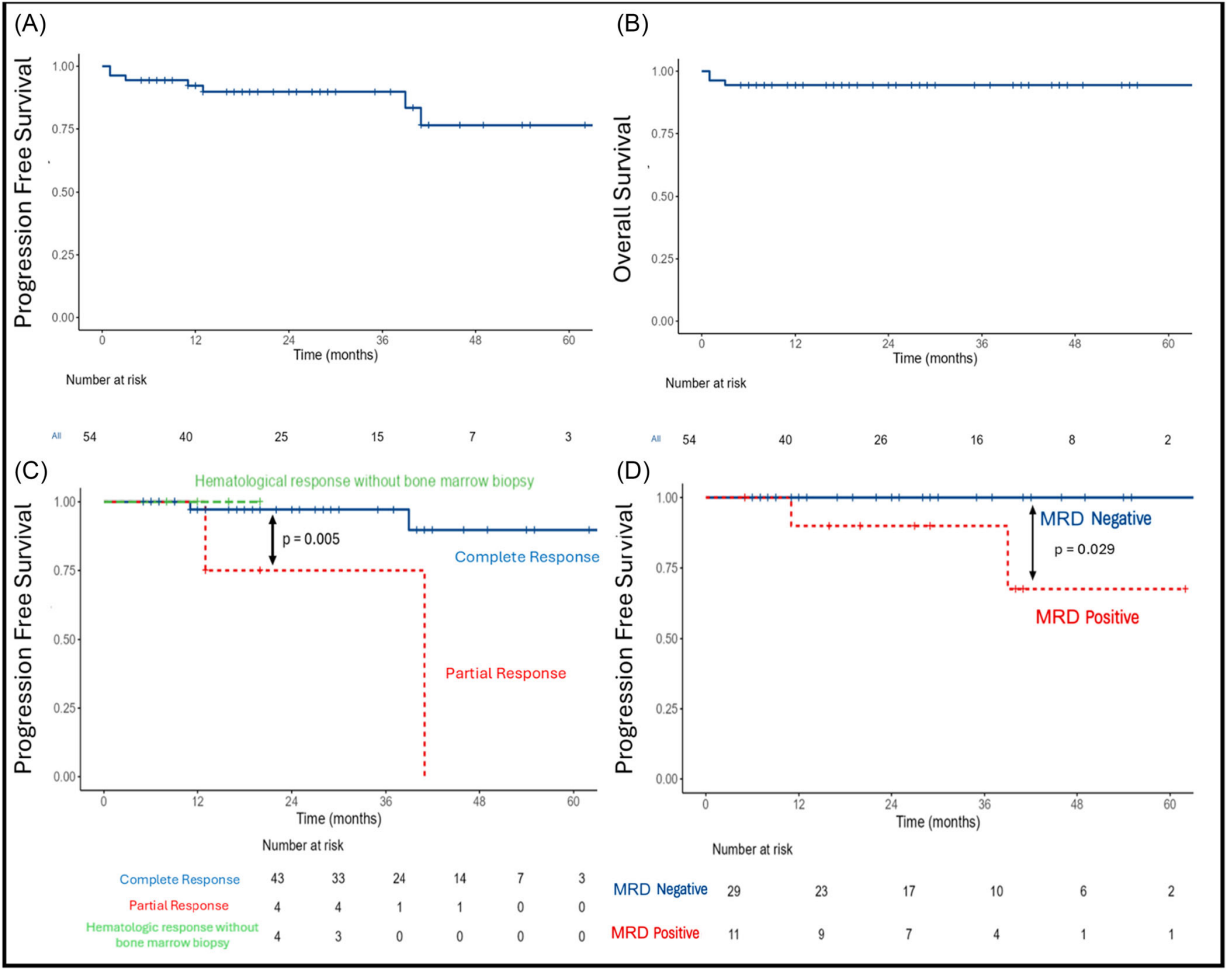
**(A)** Progression‐free survival; **(B)** overall survival; **(C)** progression‐free survival by response depth (complete response [CR], partial response [PR], and hematological response without bone marrow biopsy); and **(D)** progression‐free survival in patients with complete response by MRD status.

Again consistently with the previous trial,^18^ treatment toxicities (Table S2) were as expected for each drug, mostly of low grade, never fatal, always reversible, and mainly represented by infusion reactions related to rituximab and, for vemurafenib, by cutaneous rash, arthralgia, asymptomatic liver and pancreatic laboratory abnormalities, warts, and photosensitivity, with no myelotoxicity. Rash and arthralgia were manageable with short, low‐dose oral corticosteroid therapy. Only two premature drug discontinuations (4% of patients, 2/54) occurred due to toxicity: one patient discontinued the whole treatment due to Grade 3 rash from vemurafenib, and the other discontinued only vemurafenib (while continuing rituximab) due to Grade 3 leukoplakia from vemurafenib. In additional 34 patients, vemurafenib was briefly interrupted (*n* = 21/54 cases, 39%) for a median of 8.5 days (interquartile range [IQR] 6–18 days) and/or dose‐reduced (*n* = 24/54 cases, 44%; to 720 mg b.i.d in 12 cases; to 480 mg b.i.d. in 9; to 240 mg b.i.d. in 1 and to 240 mg daily in 2), followed by dose re‐escalation to 960 mg b.i.d. in 7/24 patients and to 720 mg b.i.d. in 11/24 patients (often supported by corticosteroids), underscoring the feasibility of proactive, steroid‐assisted toxicity management to target the planned vemurafenib exposure. Indeed, the 8‐week course of vemurafenib 960 mg b.i.d. plus eight rituximab infusions (375 mg/m^2^) was largely respected: the 51/54 evaluable patients (i.e., excluding the three early deaths) received a high relative dose intensity (RDI) of both vemurafenib (median 95%; IQR 75–100%; range 14%–180%, with one patient mistakenly receiving vemurafenib for 16 weeks instead of 8 weeks) and rituximab (median 100%; IQR 100%–100%; range 33%–100%), as well as a high dose density for vemurafenib (with the 8 planned weeks completed after a median of 56 days, IQR 56–66). The four patients with PR mostly received reduced RDI (14%, 50%, 75%, and 100% for vemurafenib; 13%, 33%, 100%, and 100% for rituximab, respectively), suggesting that keeping adequately high dose intensities might be important to obtain a deep response after such a short treatment regimen. Indeed, an association emerged between low RDI of vemurafenib (<80%) and no CR achievement among the 47 patients evaluable in this regard (chi‐square test P‐value 0.053) similar to the previous trial.^18^ In patients not achieving CR despite adequate RDI, we cannot exclude that prolonging treatment might result in conversion to CR.

Despite a shorter follow‐up (median 24 months) and a slightly less pre‐treated patient population (median of 2 prior therapies) when compared to the previous single‐center prospective HCL‐PG03 trial^18^ (37 months and 3 prior therapies, respectively), the current retrospective HCL‐PG03R study confirms, in a substantially higher number of patients (54 vs. 30,^18^ with 15/54 having active or latent infections) and in the real‐life practice of 22 Italian centers using biosimilar (rather than originator^18^) rituximab, the manageable toxicity and high efficacy (CR rate 86%; MRD‐negativity rate 62%; 90% PFS at 24 months) of a very short chemotherapy‐free treatment combining 8 weeks of vemurafenib standard dose (960 mg b.i.d.) with 8 rituximab infusions. Our study also reinforces the notable added value of rituximab on top of vemurafenib.^17^, ^18^ Notably, this drug regimen entailing biosimilar rituximab is also relatively cheap (~7300 euro/patient in the Italian health system) and, although its registration at the EMA or FDA is not being pursued by a disinterested pharmaceutical company, it was autonomously approved in August 2023 by the Italian drug agency AIFA for reimbursements in patients with HCL in the ≥3rd line setting as well as in those refractory to, or unfit for, chemotherapy. We are now testing this regimen in the general population of previously untreated HCL through an academic, multicenter, randomized clinical trial against the chemotherapy‐based standard of care (EUCT number 2024‐520119‐41‐00).

## AUTHOR CONTRIBUTIONS

**Luca De Carolis**: Investigation. **Monia Capponi**: Investigation. **Andrea Bernardelli**: Methodology; data curation; investigation; writing—original draft; project administration; formal analysis; software. **Andrea Visentin**: Investigation. **Gianna Maria D'Elia**: Investigation. **Simone Ferrero**: Investigation. **Michele Cimminiello**: Investigation. **Azzurra Romeo**: Investigation. **Barbara Pocali**: Investigation. **Luciana Morino**: Investigation. **Alessandro Sanna**: Investigation. **Ombretta Annibali**: Investigation. **Francesca Ricci**: Investigation. **Flavio Falcinelli**: Investigation. **Alessandro Mancini**: Investigation. **Francesco Angotzi**: Investigation. **Anna Maria Frustaci**: Investigation. **Nilla Maschio**: Investigation. **Caterina Stelitano**: Investigation. **Alessandro Gozzetti**: Investigation. **Riccardo Moia**: Investigation. **Maura Nicolosi**: Investigation. **Silvia Trappolini**: Investigation. **Valerio Leotta**: Investigation. **Alessandro Pulsoni**: Investigation. **Robin Foà**: Investigation. **Carlo Visco**: Investigation. **Roberta Murru**: Investigation. **Jacopo Olivieri**: Investigation. **Livio Trentin**: Investigation. **Brunangelo Falini**: Investigation. **Enrico Tiacci**: Conceptualization; data curation; methodology; investigation; validation; formal analysis; supervision; writing—review and editing; visualization; project administration; resources; funding acquisition.

## CONFLICT OF INTEREST STATEMENT

The authors declare no conflicts of interest.

## ETHICS STATEMENT

Approved by the Ethics Committee of Perugia (CER N registry number 4240/19).

## FUNDING

Open access publishing facilitated by Universita degli Studi di Perugia, as part of the Wiley ‐ CRUI‐CARE agreement.

This work has been supported by Associazione Italiana per la Ricerca sul Cancro/AIRC “Metastasis 5‐per‐mille” grant no. 21198 to E.T. and B.F., Hairy Cell Leukemia Foundation ‐ Leukemia & Lymphoma Society (HCL2025 Initiative ‐ Translational Research grant no. HCL8029‐22 to E.T.), and Fondazione Perugia (Bando Ricerca 2024 – Progetto di ricerca innovativo 22870/2024.0362).

## Supporting information

Supporting Information.

## Data Availability

The data that support the findings of this study are available from the corresponding author upon reasonable request.
