# Supplementary material for: Real‐life efficacy and safety of vemurafenib plus rituximab in relapsed or refractory hairy‐cell leukemia: A multicenter Italian retrospective study (HCL‐PG03R)
Source: Hemasphere. 2025 Nov 14;9(11):e70255. doi: 10.1002/hem3.70255 (PMC12616395; doi:10.1002/hem3.70255)
Supplement: Supplementary file 1 — Supporting Information. [file HEM3-9-e70255-s001.docx]

**Table S1. Individual patient characteristics**

| **Pt#, Sex, Age** | **Previous therapies** | **Blood counts and spleen size** | | | | **Relative dose intensity** | | **No. of days needed to complete the 8 weeks of vemurafenib (dose density)** | **Response to vemurafenib+ rituximab** | **MRD status of CR** | **PFS**  **(months)** |
| --- | --- | --- | --- | --- | --- | --- | --- | --- | --- | --- | --- |
|  |  | *Hb*  *g/dl* | *Plts*  *x 10^3^/mm^3^* | *Neut /mm^3^* | *Spleen*  *(cm)* | *Vemurafenib* | *Ritux.* |  |  |  |  |
| 1, M, 68 | DCF, CDA, DCF+R | 10.2 | 67000 | 860 | 14.5 | 50% | 33% | 71 | PR | n.a. | 13 Rel |
| 2, M, 67 | DCF, CDA | 13.2 | 41000 | 580 | 11 | 100% | 100% | 56 | HR (without BMB) | n.a. | 16 |
| 3, F, 39 | CDA^ | 12.8 | 59000 | 760 | 11 | 85% | 100% | 56 | HR (without BMB) | n.a. | 12 |
| 4, M, 63 | CDA, DCF | 12.8 | 57000 | 670 | 15 | 100% | 100% | 56 | CR | POS | 62 |
| 5, M, 51 | CDA, CDA, R+DCF | 9.4 | 40000 | 600 | 13.5 | 100% | 100% | 56 | CR | n.a. | 9 |
| 6, M, 58 | CDA, CDA | 13.4 | 47000 | 900 | 13 | 14% | 13% | 8 | PR | POS | 41 Rel |
| 7, M, 56 | CDA^ | 12.9 | 105000 | 1180 | 17 | 79% | 100% | 63 | CR | POS | 27 |
| 8, M, 60 | IFN, CDA, CDA, INF, R, R, INF, DCF*, R, SPL, DABRA | 10.9 | 181000 | 1210 | n.a. | 100% | 100% | 56 | CR | NEG | 42 |
| 9, M, 48 | CDA* | 7.4 | 50000 | 410 | 15.6 | 100% | 100% | 56 | CR | NEG | 64 |
| 10, M, 77 | CDA^ | 14.8 | 64000 | 1710 | 12.8 | 97% | 50% | 61 | n.a. (D) | n.a. | 3 |
| 11, M, 48 | CDA* | 12.4 | 69000 | 1090 | 17 | 88% | 100% | 56 | CR | NEG | 28 |
| 12, M, 64 | DCF, IFN, R, DCF, IFN, CDA | 12.4 | 65000 | 780 | 16 | 100% | 100% | 60 | CR | NEG | 54 |
| 13, M, 56 | DCF, CDA+R | 11.6 | 73000 | 640 | 9.5 | 100% | 100% | 56 | CR | POS | 29 |
| 14, M, 61 | IFN, R, R, R, R, R, R, R*, R+FLU, DCF | 12.4 | 69000 | 1630 | 16 | 100% | 100% | 58 | CR | NEG | 30 |
| 15, M, 77 | IFN, IFN, IFN, CDA, R | 13.1 | 70000 | 1320 | 15 | 81% | 100% | 57 | CR | NEG | 17 |
| 16, M, 62 | DCF^ | 8.7 | 73000 | 1020 | 7 | 71% | 100% | 136 | CR | NEG | 37 |
| 17, M, 56 | CDA, CDA | 13.4 | 69000 | 1220 | 12.7 | 74% | 100% | 118 | CR | NEG | 19 |
| 18, F, 45 | CDA^ | 9.5 | 87000 | 830 | 12.5 | 100% | 100% | 56 | CR | NEG | 49 |
| 19, M, 58 | CDA, CDA | 13.7 | 66000 | 670 | 12.7 | 75% | 100% | 52 | CR | POS | 41 |
| 20, M, 80 | / | 9.6 | 108000 | 940 | 15.5 | 79% | 100% | 56 | CR | POS | 40 |
| 21, M, 48 | CDA* | 10.1 | 101000 | 500 | 12 | 100% | 100% | 55 | CR | NEG | 30 |
| 22, M, 42 | CDA* | 11.7 | 150000 | 800 | 9.5 | 100% | 100% | 63 | CR | NEG | 49 |
| 23, M, 65 | / | 9.1 | 43000 | 240 | 10 | 59% | 100% | 58 | CR | NEG | 8 |
| 24, F, 78 | CDA, CDA* | 10.8 | 40000 | 1020 | 9.8 | 81% | 100% | 62 | CR | NEG | 25 |
| 25, F, 66 | CDA^ | 10.8 | 69000 | 1040 | 13.5 | 81% | 100% | 57 | CR | NEG | 29 |
| 26, M, 75 | Spl, IFN, CDA, CDA, FLU+R, CDA, VEMU+Cobimetinib+ Obinutuzumab | 12.7 | 93000 | 2150 | n.a. | 100% | 100% | 55 | CR | POS | 11 Rel |
| 27, M, 66 | IFN, R, CDA, VEMU, VEMU*, IFN | 10.6 | 57000 | 450 | 16 | 100% | 100% | 56 | CR | POS | 29 |

| 28, M, 51 | CDA, IFN | 8.5 | 65000 | 1770 | 22 | 63% | 43% | 35 | n.a. (D) | n.a. | 1 |
| --- | --- | --- | --- | --- | --- | --- | --- | --- | --- | --- | --- |
| 29, F, 50 | IFN, CDA | 11.9 | 710000 | 1250 | 16 | 97% | 100% | 56 | CR | NEG | 19 |
| 30, M, 55 | DCF, IFN, IFN, CDA, R | 11.9 | 170000 | 1410 | 10.3 | 22% | 100% | 15 | CR | NEG | 13 |
| 31, M, 53 | DCF, DCF, IFN, IFN, IFN | 8.1 | 36000 | 1020 | 20 | 101% | 100% | 68 | CR | NEG | 22 |
| 32, M, 56 | CDA, CDA | 9.4 | 75000 | 800 | 15 | 180% | 100% | 110 | CR | NEG | 9 |
| 33, M, 64 | CDA, CDA | 8.9 | 51000 | 400 | 14 | 66% | 100% | 76 | CR | NEG | 24 |
| 34, M, 50 | CDA, CDA | 10.3 | 56.000 | 860 | 16.7 | 79% | 100% | 81 | CR | NEG | 7 |
| 35, F, 58 | CDA^ | 10.8 | 120.000 | 500 | n.a. | 26% | 100% | 35 | HR (without BMB) | n.a. | 8 |
| 36, M, 54 | IFN, CDA, CDA | 12.8 | 104.000 | 750 | 10 | 80% | 100% | 56 | CR | NEG | 41 |
| 37, M, 53 | CDA, CDA, VEMU+ Cometinib, R | 12.4 | 78.000 | 600 | 13.4 | 65% | 100% | 70 | CR | n.a. | 18 |
| 38, M, 59 | CDA, CDA, IFN, VEMU+R | 10.1 | 79.000 | 1000 | n.a. | 75% | 100% | 83 | PR | n.a. | 20 |
| 39, M, 57 | Spl, IFN, CDA, Zanu. | 8.6 | 134000 | 1310 | n.a. | 100% | 100% | 56 | CR | NEG | 12 |
| 40, M, 69 | DCF, CDA | 9.7 | 21000 | 340 | 16.5 | 100% | 100% | 56 | CR | POS | 39 Rel |
| 41, M, 42 | CDA*, R+CDA | 13 | 52000 | 760 | 14.5 | 100% | 100% | 56 | CR | NEG | 66 |
| 42, M, 56 | CDA* | 6.6 | 21000 | 240 | n.a. | 100% | 100% | 56 | CR | NEG | 11 |
| 43, M, 56 | DCF, CDA, IFN, CDA, R*, CDA* | 6.4 | 34000 | 160 | 15 | 75% | 37% | 44 | n.a. (D) | n.a. | 1 |
| 44, M, 55 | DCF, BENDA, R, R* | 10.6 | 42000 | 850 | 21.5 | 93% | 100% | 72 | CR | NEG | 46 |
| 45, M, 63 | CDA^ | 13.8 | 78000 | 1060 | 13.5 | 100% | 100% | 59 | CR | NEG | 6 |
| 46, F, 35 | CDA* | 12.8 | 114000 | 1100 | 15 | 41% | 100% | 70 | CR | NEG | 55 |
| 47, M, 45 | CDA^ | 14.7 | 77000 | 1180 | 23 | 100% | 100% | 56 | CR | NEG | 11 |
| 48, M, 53 | CDA^ | 8.8 | 97000 | 690 | n.a. | 100% | 100% | 67 | CR | NEG | 35 |
| 49, M, 53 | CDA, DCF | 12.1 | 42000 | 350 | 16.4 | 94% | 100% | 56 | CR | n.a. | 6 |
| 50, M, 44 | CDA^ | 7.3 | 34000 | 410 | 13 | 100% | 100% | 56 | HR (without BMB) | n.a. | 20 |
| 51, M, 62 | CDA, DCF | 11.1 | 46000 | 810 | 16 | 100% | 100% | 56 | CR | POS | 16 |
| 52, M, 55 | DCF*, IFN, CDA*, IFN, IFN, Moxetumomab pasudotox, IFN | 8.8 | 73000 | 1230 | 14 | 100% | 100% | 77 | PR | n.a. | 13 |
| 53, M, 57 | CDA+R^ | 13.8 | 55000 | 1100 | 16 | 91% | 100% | 56 | CR | POS | 5 |
| 54, M, 68 | CDA^ | 13.2 | 72000 | 1200 | 12.5 | 75% | 100% | 77 | CR | POS | 20 |

*No response to the indicated therapy. ^Patients no. 3-7-10-16-18-25-35-45-47-48-50-53-54 were treated in the second line with vemurafenib plus rituximab at the investigator discretion due to relapse occurring respectively at 49-55-48-50-25-48-101-48-32-28-5-56-88 months after cladribine or pentostatin. BENDA, Bendamustine; CDA, cladribine; CR, complete response; D, Death before response evaluation; DCF, pentostatin; F, female; FLU, fludarabine; Hb, hemoglobin; HR (without BMB), hematologic response without bone marrow biopsy; IFN, interferon-α; M, male; MRD, minimal residual disease status; n.a., not available; NEG, negative; Neut, neutrophils; PFS, Progression-Free Survival; Plts, platelets; POS, positive; PR, Partial Response; Pt#, patient identification number.; R, Rituximab; Rel, relapse; Spl, splenectomy; Spleen (cm), longest spleen diameter measured radiologically.

***Table S2: Drug-related Adverse Events in the 54 enrolled patients (grading according to CTCAEv5***

| **Rituximab-related adverse event** | **Grade 1-2** | **Grade 3** | **Grade 4** | **Total** |
| --- | --- | --- | --- | --- |
| *Number of patients with event (percent)* | | | | |
| **Infusion-related reaction** | 9 (17) | 2 (4) | / | 11 (20) |
| **Transient neutropenia** | / | 1^#^ (2) | 1^#^ (2) | 2 (4) |
| **Herpes Simplex** | 1 (2) | / | / | 1 (2) |

| **Vemurafenib-related adverse event** | **Grade 1-2** | **Grade 3** | **Grade 4** | **Total** |
| --- | --- | --- | --- | --- |
| *Number of patients with event (percent)* | | | | |
| **Cutaneous rash** | 18 (33) | 5 (9) | / | 23 (43) |
| **Hyperbilirubinemia** | 18 (33) | 2^#^ (4) | / | 20 (37) |
| **Arthralgia or arthritis** | 16^£^ (30) | 2^ç^ (4) | / | 18 (33) |
| **Lipase or amylase increase** | 6 (11) | 3^#^ (6) | / | 9 (17) |
| **Skin papilloma or warts** | 9 (17) | / | / | 9 (17) |
| **GGT or ALP increase** | 9 (17) | / | / | 8 (17) |
| **Photosensitivity** | 8 (15) | / | / | 8 (15) |
| **Creatinine increase** | 5 (9) | / | / | 5 (9) |
| **ALT or AST increase** | 4 (7) | / | / | 4 (7) |
| **Fever** | 3 (5) | / | 1^&^ (2) | 4 (7) |
| **Headache** | 3 (5) | 1 (2) | / | 4 (7) |
| **Hypophosphatemia** | / | 3^#^ (5) | / | 3 (5) |
| **Hyperkeratosis** | 3 (5) | / | / | 3 (5) |
| **Asthenia or fatigue** | 1 (1) | 2 (4) | / | 3 (5) |
| **Nausea, anorexia or dyspepsia** | 2 (4) | / | / | 2 (4) |
| **Pain in hands and/or feet** | 2 (4) | / | / | 2 (4) |
| **Leukoplakia** | / | 1 (2) | / | 1 (2) |
| **QTc-interval prolongation** | 1 (2) | / | / | 1 (2) |
| **Suspected keratoacanthoma** | 1 (2) | / | / | 1 (2) |
| **Basal cell carcinoma** | 1 (2) |  | / | 1 (2) |
| **Abdominal pain** | 1 (2) | / | / | 1 (2) |
| **Neutropenia** | 1 (2) | / | / | 1 (2) |
| **Alopecia** | 1 (2) | / | / | 1 (2) |

***^#^*** *Asymptomatic****^£^*** *13 arthralgia, 3 arthritis****^ç^*** *2 arthralgia****^&^*** *Possibly related to vemurafenib (fever developed after 30 days of vemurafenib 960 mg bid in the absence of neutropenia, lasted 6 days, was managed with levofloxacin and vemurafenib suspension for 6 days, and did not recur upon resuming vemurafenib at 720 mg bid*
